# Lactylation-Related Gene Signature Effectively Predicts Prognosis and Treatment Responsiveness in Hepatocellular Carcinoma

**DOI:** 10.3390/ph16050644

**Published:** 2023-04-25

**Authors:** Zhe Cheng, Huichao Huang, Maoyu Li, Xujun Liang, Yuying Tan, Yongheng Chen

**Affiliations:** 1 Department of Oncology, NHC Key Laboratory of Cancer Proteomics, Laboratory of Structural Biology, National Clinical Research Center for Geriatric Disorders, Xiangya Hospital, Central South University, Changsha 410008, China; tearsacz@csu.edu.cn (Z.C.);; 2 Department of Infectious Disease, NHC Key Laboratory of Cancer Proteomics, Laboratory of Structural Biology, National Clinical Research Center for Geriatric Disorders, Xiangya Hospital, Central South University, Changsha 410008, China

**Keywords:** hepatocellular carcinoma, lactylation-related genes, prognostic model, tumor immune environment, treatment response, protein lactylation

## Abstract

Background: Hepatocellular carcinoma (HCC) is a malignant tumor associated with high morbidity and mortality. Therefore, it is of great importance to develop effective prognostic models and guide clinical treatment in HCC. Protein lactylation is found in HCC tumors and is associated with HCC progression. Methods: The expression levels of lactylation-related genes were identified from the TCGA database. A lactylation-related gene signature was constructed using LASSO regression. The prognostic value of the model was assessed and further validated in the ICGC cohort, with the patients split into two groups based on risk score. Glycolysis and immune pathways, treatment responsiveness, and the mutation of signature genes were analyzed. The correlation between PKM2 expression and the clinical characteristics was investigated. Results: Sixteen prognostic differentially expressed lactylation-related genes were identified. An 8-gene signature was constructed and validated. Patients with higher risk scores had poorer clinical outcomes. The two groups were different in immune cell abundance. The high-risk group patients were more sensitive to most chemical drugs and sorafenib, while the low-risk group patients were more sensitive to some targeted drugs such as lapatinib and FH535. Moreover, the low-risk group had a higher TIDE score and was more sensitive to immunotherapy. PKM2 expression correlated with clinical characteristics and immune cell abundance in the HCC samples. Conclusions: The lactylation-related model exhibited robust predictive efficiency in HCC. The glycolysis pathway was enriched in the HCC tumor samples. A low-risk score indicated better treatment response to most targeted drugs and immunotherapy. The lactylation-related gene signature could be used as a biomarker for the effective clinical treatment of HCC.

## 1. Introduction

As the most common pathological subtype of primary liver cancer, hepatocellular carcinoma (HCC) diagnosis and treatment have always been under the spotlight. Primary liver cancer was the third leading cause of cancer-related death worldwide in 2020. Liver cancer ranks fifth in global incidence according to global cancer statistics [1]. Primary liver cancer includes HCC (approximately 80% of cases), intrahepatic cholangiocarcinoma (10–15%), and some rare types. HCC can be treated with surgery if diagnosed early. Other treatments include ablation therapy, embolization therapy, radiation therapy, chemotherapy, targeted therapy, and immunotherapy. However, HCC may develop resistance toward these chemicals and targeted drugs, which can attenuate treatment efficacy [2,3]; in addition, the high heterogeneity of HCC has become an obstacle in HCC treatment [4]. Therefore, developing an effective prognostic model that is useful in selecting proper treatment approaches and drugs for HCC treatment is an urgent issue.

Protein lactylation is a novel form of protein posttranslational modification first reported in 2019 that involves the conjugation of lactate molecules to lysine residues [5]. The Warburg effect describes abnormal anaerobic glycolysis and excessive lactate accumulation [6]. It has been reported that metabolic reprogramming is rather common in HCC occurrence, which includes changes in glucose, fatty acid synthesis, and metabolism. Enhanced anaerobic glycolysis is one of the hallmarks of HCC [7]. For instance, it has been revealed that low-affinity hexokinase 4 (HK4) is switched to high-affinity HK2 in HCC occurrence, which results in higher glycolytic rates [8]. In proliferating cells including HCC cells, the specific pyruvate kinase M2 (PKM2) isoform is highly expressed and increases the intensity of anaerobic glycolysis [9]. Metabolic reprogramming also includes the diversion of the glycolysis pathway, which fulfills the needs of tumor progression. Tumor cell proliferation requires abundant ribose 5-phosphate, and nicotinamide adenine nucleotide phosphate (NADPH) provides energy for the antioxidant system that resists oxidative stress in tumor cells. Glucose-6-phosphate isomerase shunts glucose to the pentose phosphate pathway to produce NADPH and pentose in HCC, breast cancer, and colorectal cancers [10,11,12]. 

Research has found that protein lactylation widely exists in various human normal tissues and cancer tissues including HCC tissues and cell lines [13,14,15]. Studies have further shown that lactylation is closely related to cell energy metabolism and cancer immunity. Wang et al. reported that several sites on PKM2 can be lactylated and that specific K62 lactylation is responsible for the functional regulation of PKM2. PKM2 lactylation increases the enzymatic activity of PKM2 and reduces the tetramer-to dimer transition and nuclear distribution, resulting in a feedback pathway that inhibits glycolysis [16]. Adenylate kinase 2 (AK2) is the key enzyme that catalyzes the transfer of phosphate groups between ATP and adenosine monophosphate (AMP) and generates adenosine diphosphate (ADP). Yang and coworkers found that AK2 lactylation significantly reduced AK2 enzymatic activity, resulting in energy disorder in HCC cells [14]. Based on these inspiring findings, we determined that these lactylation-related genes were closely connected to cell metabolism, especially glycolysis. Given the major role lactylation plays in cancer immunity, HCC protein lactylation is bound to make a difference in tumor cancer immunity. HCC immunotherapy takes effect mainly by activating immune checkpoints including programmed death-1 (PD-1), its ligand PD-L1, and cytotoxic T-lymphocyte-associated protein 4 (CTLA-4). Immune checkpoint inhibition (ICI) using corresponding antibodies has been broadly researched in the field [17]. 

Here, we collected the lactylation-related data published thus far and systematically analyzed the expression levels of these genes in different databases to identify a gene signature that predicts patient prognosis. The least absolute shrinkage and selection operator (LASSO) is a common method used to establish prognostic models. It provides satisfactory prediction accuracy and interpretability [18]. Glycolysis pathway enrichment of the samples and mutation of the model genes were evaluated. The drug sensitivity and tumor immune dysfunction and exclusion (TIDE) score were carefully analyzed to provide valuable clinical instructions for HCC patients. In addition, we performed survival, clinical relevance, and immune cell relevance analyses on one of the signature’s key genes. Remarkably, a lactylation-related gene signature that effectively predicts both prognosis and treatment responsiveness in HCC has not been reported to date. This study represents a significant contribution to the field by filling this gap in the current knowledge.

## 2. Results

### 2.1. Identifying Prognostic Lactylation-Related Genes in HCC

**Figure 1:**
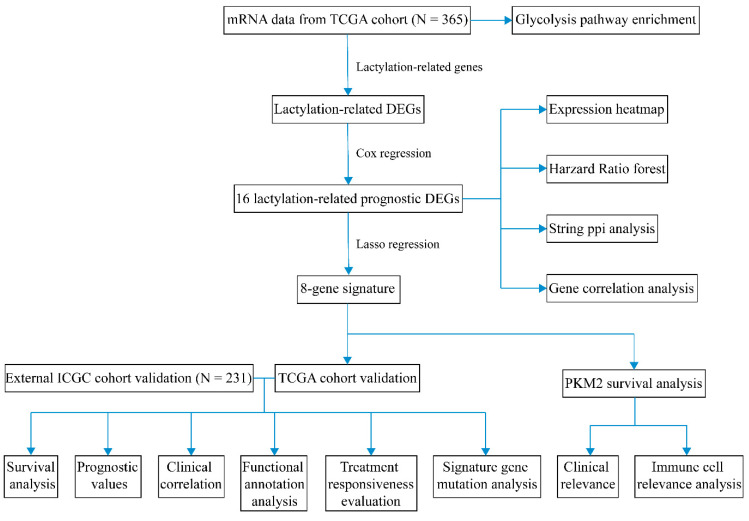
Study workflow diagram.

**Figure 2:**
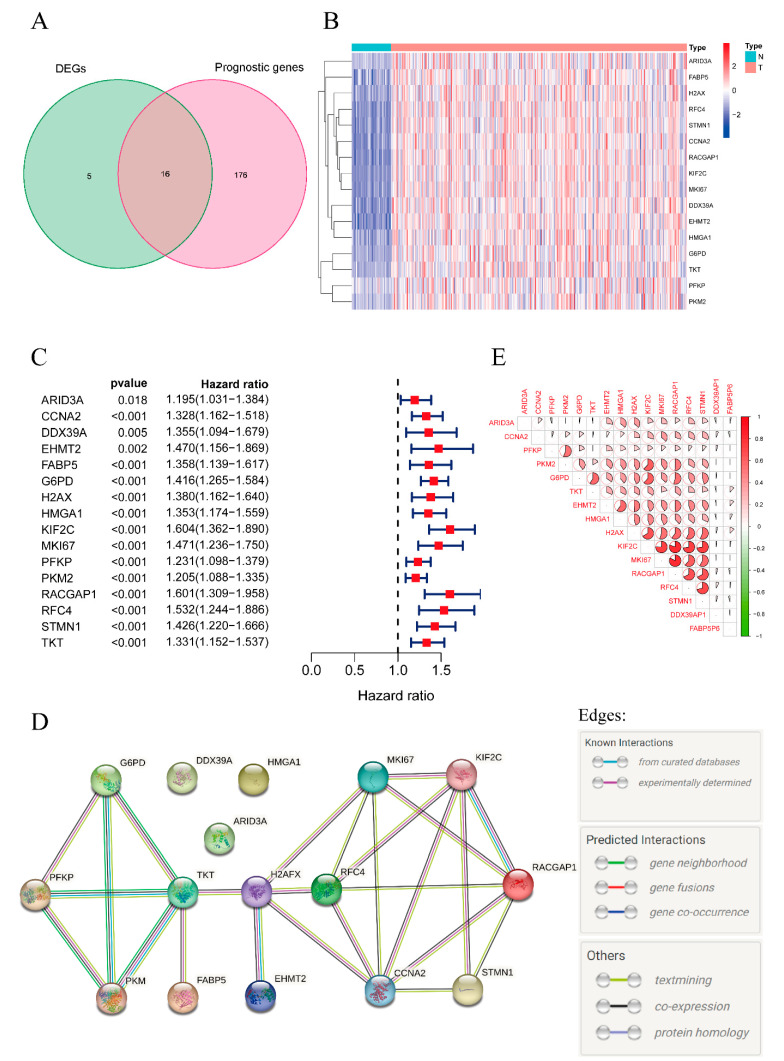
Identification of prognostic lactylation-related genes in HCC. (**A**) Venn diagram showing the quantities of the prognostic lactylation-related genes. (**B**) Heatmap presenting the expression levels of the prognostic lactylation-related genes (Type N: adjacent tissues; T: tumor tissues). (**C**) Forest plot of prognostic lactylation-related DEGs. (**D**) PPI network showing known and predicted interactions of proteins and genes among the prognostic lactylation-related DEGs. (**E**) The results of the gene correlation analysis between intersectional genes.

Figure 1 displays the overall procedure of the study. The clinical and pathological characteristics of HCC patients in the TCGA and ICGC cohorts are listed in Table 1. Data on the prevalence of hepatitis viral infection in the TCGA cohort are provided in Supplementary Tables S2 and S3. Patients with HBV DNA, HCV RNA, genotype, or antibody were identified. There were 223 patients infected with HBV and 133 patients infected with HCV. The mRNA data of 365 cancer tissues and 50 paired adjacent tissues with corresponding clinical information were obtained from the TCGA database. Differentially expressed genes (DEGs) and prognostic lactylation-related genes were screened out. Eventually, we identified 16 lactylation-related prognostic DEGs: ARID3A/DRIL1, CCNA2, DDX39A/DDX39, EHMT2/KMT1C, FABP5, G6PD, H2AX/H2AFX, HMGA1, KIF2C/KNSL6, MKI67, PFKP, PKM2, RACGAP1/CYK4, RFC4, STMN1, and TKT. The quantities of prognostic genes and DEGs are shown in a Venn diagram (Figure 2A). A heatmap was constructed to show the expression levels of these lactylation-related DEGs (Figure 2B). As shown in the heatmap, the 16 genes all exhibited higher expression levels in cancer tissues than in the adjacent tissues. A forest map was also constructed and showed that these genes were all risk genes with different hazard ratios (HRs) (Figure 2C). Then, we performed PPI analysis using STRING. The PPI network in Figure 2D indicates that most of the proteins encoded by these genes were closely connected in an intricate manner. Notably, four genes largely involved in glucose metabolism and glycolysis, PFKP, PKM2, G6PD and TKT, displayed extensive associations in the PPI network. The PPI network showed currently known interactions and predicted interactions between the proteins encoded by the prognostic DEGs. Figure 2E shows the results of the gene correlation analysis. Strong positive correlations at the transcriptome level were observed between the following genes: KIF2C and MKI67, RACGAP1, RFC4, STMN1; MKI67 and RACGAP1; PKM2 and PFKP.

### 2.2. Constructing a Prognostic Model in the TCGA Cohort

**Figure 3:**
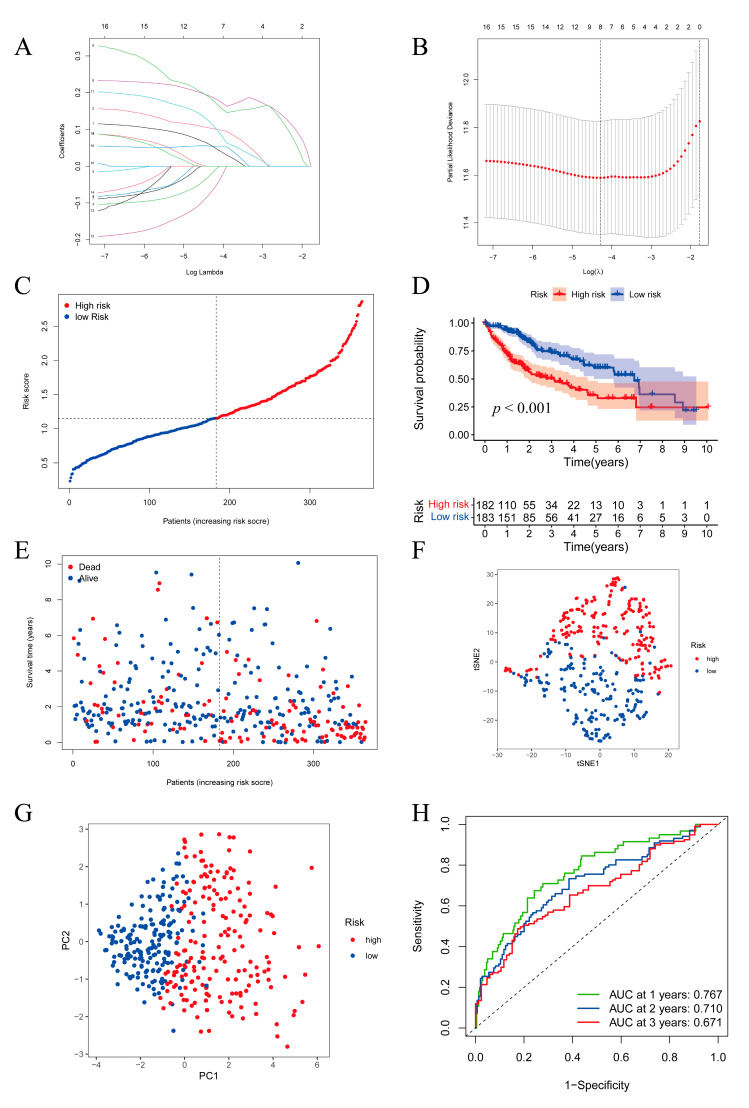
Construction of the prognostic signature in the TCGA cohort. (**A**) The gene coefficient profiles determined by LASSO regression. (**B**) The partial likelihood deviance plotted with log (λ). (**C**) Distribution of the risk score in the TCGA cohort. (**D**) Kaplan–Meier survival curves of the HCC overall survival in the TCGA cohort. (**E**) Distribution of survival status with an increasing risk score in the TCGA cohort. (**F**) T-SNE analysis of patients in the TCGA cohort. (**G**) PCA of patients in the TCGA cohort. (**H**) ROC curves showing that the 8-gene prognostic signature had satisfactory predictive efficacy in the TCGA cohort.

LASSO Cox regression was performed to construct the prognostic model according to the expression levels of the 16 intersectional genes. An eight-gene model was obtained based on the optimal value of λ (Figure 3A), and the lowest partial likelihood deviance is shown in Figure 3B. The risk score model was as follows: Risk score = (0.0552 × expression level of ARID3A) + (0.1030 × expression level of CCNA2) + (0.1877 × expression level of G6PD) + (0.1814 × expression level of KIF2C) + (0.1142 × expression level of PFKP) + (0.05427 × expression level of TKT) − (0.0192 × expression level of DDX39A) − (0.0676 × expression level of PKM2). Based on the calculated risk score of each patient, patients in the TCGA cohort were divided into high- and low-risk groups (Figure 3C). As anticipated, the Kaplan–Meier survival curves showed that the survival rates were lower in the high-risk group than in the low-risk group (*p* = 6.915 × 10^−7^) (Figure 3D). Consistently, patients in the high-risk group had shorter survival times as the risk score increased (Figure 3E). We further conducted t-distributed stochastic neighbor embedding (t-SNE), principal component analysis (PCA), and ROC analysis to assess the prognostic model in multiple dimensions. As shown in Figure 3F,G, the model clearly divided the patients into two subgroups. The areas under the curve (AUCs) were 0.767 at 1 year, 0.710 at 2 years and 0.671 at 3 years, indicating that the model had satisfactory predictive efficacy in HCC (Figure 3H).

### 2.3. Validating the Prognostic Lactylation-Related Signature in the ICGC Cohort

The prognostic value of the gene signature was validated in the ICGC cohort. In total, 231 patients were separated into high- and low-risk groups based on the risk score calculated by the formula (Figure S1A). The PCA and t-SNE analysis confirmed that the risk model clearly divided the patients into two groups (Figure S1B,C). The Kaplan–Meier curve also indicated that patients in the high-risk group had a lower survival probability than patients in the low-risk group (*p* = 0.003129) (Figure S1D). Similarly, a consistent tendency of survival time and risk scores was observed in the two subgroups, which indicated that the quantity of the patients alive decreased in the high-risk group as the risk score increased (Figure S1E). Figure S1F shows the ROC curve in the ICGC cohort, and the risk score model exhibited a fair prognostic value as the AUCs reached 0.742 at 1 year, 0.754 at 2 years, and 0.758 at 3 years. The expression levels of alpha-fetoprotein (AFP) were evaluated in the high- and low-risk groups, and the results showed that the AFP expression level was significantly higher in the high-risk group than in the low-risk group (*** *p* < 0.001), as shown in Figure S3 in the Supplementary Materials. As AFP is a widely recognized HCC biomarker, this result indicated that the prognostic model presented coherent predictive efficacy with AFP expression. These inspiring results show that the prognostic model could well divide the patients into two subgroups and predict the prognosis of the patients effectively.

### 2.4. Independent Prognostic Value of the Lactylation-Related Eight-Gene Signature

**Figure 4:**
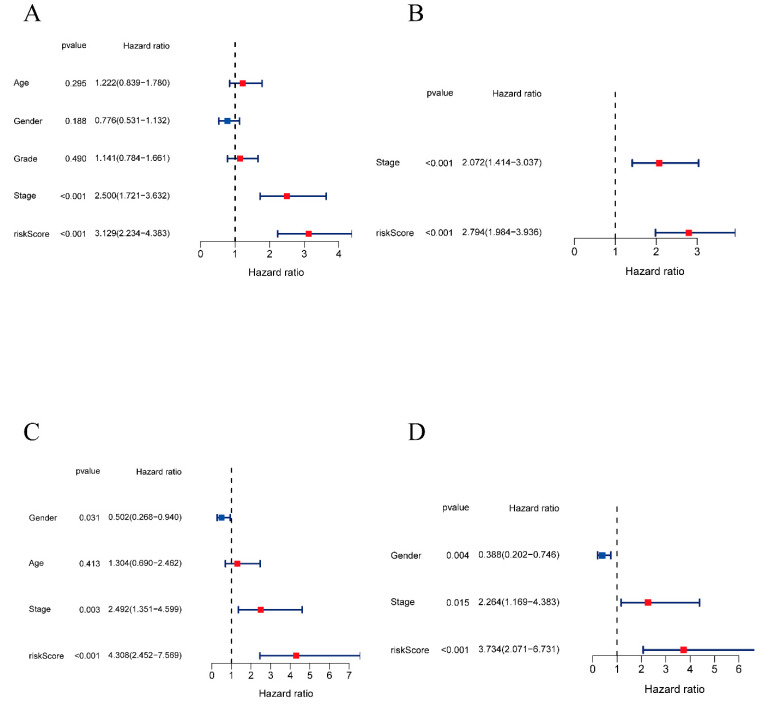
Univariate and multivariate independent prognostic analyses. (**A**) Univariate independent prognostic analysis in the TCGA cohort. (**B**) Multivariate independent prognostic analysis in the TCGA cohort. (**C**) Univariate independent prognostic analysis in the ICGC cohort. (**D**) Multivariate independent prognostic analysis in the ICGC cohort.

**Figure 5:**
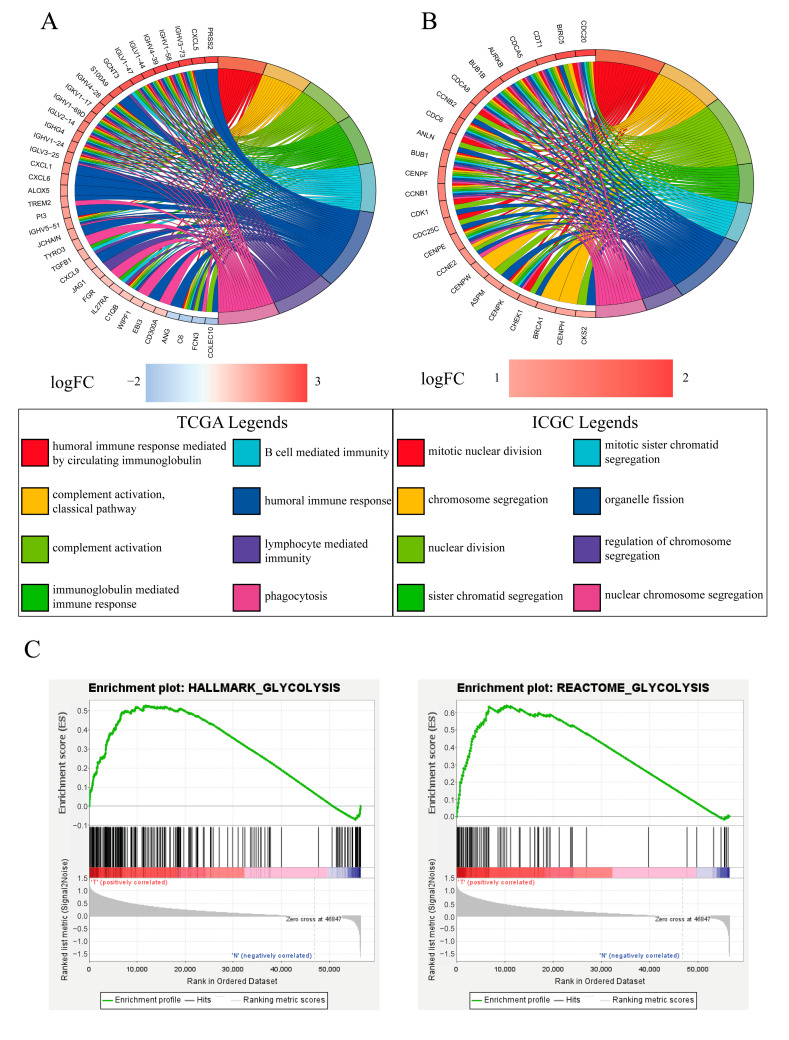
Functional annotation analysis and glycolytic pathway GSEA. (**A**) GO annotation analysis in the TCGA cohort. (**B**) GO annotation analysis in the ICGC cohort. (**C**) GSEA of glycolysis pathway enrichment among the tumor and adjacent tissues.

To measure the independent prognostic value and clinical correlations of the gene signature, univariate and multivariate independent prognostic analyses were conducted. The analyses were processed using the “survival” R package. The age threshold was set at 65 years old. For grade, patients with Grades 1 and 2 were set as the control group, while those with Grades 3 and 4 were set as the experimental group. Similarly, patients in different stages were divided into a control group and an experimental group. In the TCGA cohort, the univariate analysis revealed that both the stage and risk score were significantly related to the overall survival time, and the hazard ratio for risk score was 3.129 (95% CI: 2.234–4.383, *p* value < 0.001) (Figure 4A). The multivariate analysis confirmed the clinical correlation with a hazard ratio of 2.794 (95% CI: 1.984–3.936, *p* value < 0.001) (Figure 4B). Furthermore, in the ICGC cohort, the risk score model also indicated that the risk model was a credible independent prognostic factor for OS in HCC (HR = 4.308, 95% CI: 2.452–7.569, *p* value < 0.001; HR = 3.734, 95% CI: 2.071–6.731, *p* value < 0.001) (Figure 4C and Figure 5D). Collectively, these data show that the 8-gene prognostic signature had a good correlation with clinical prognosis and could predict the clinical outcome independently. 

### 2.5. Functional Annotation and Glycolysis Pathway GSEA

To determine the differential functional pathways between the high- and low-risk groups divided by the gene signature, we performed GO annotation analyses in both the TCGA and ICGC databases. In the TCGA cohort, immune-related processes were largely enriched such as the humoral immune response, lymphocyte-mediated immunity, and immunoglobulin-mediated immune response (Figure 5A), while DNA replication and transcription including nuclear division, mitotic nuclear division, and chromosome segregation were enriched in the ICGC cohort (Figure 5B). As protein lactylation is closely associated with glycolysis, we conducted glycolysis pathway GSEA among the tumor tissues and adjacent tissues. As expected, the GSEA results indicated that the glycolysis pathway was largely enriched in cancer tissues compared with adjacent tissues (Figure 5C).

### 2.6. TIME Landscape Analysis and Treatment Responsiveness Evaluation

As the GO analysis indicated pathway enrichment related to the tumor immune microenvironment (TIME), we conducted ssGSEA to evaluate immune cell infiltration and immune functions in the TCGA and ICGC cohorts. The abundance of immune cells was significantly higher in the high-risk group than in the low-risk group in both the TCGA and ICGC cohorts. In the TCGA cohort, aDC, iDC, and Tfh, Th1, Th2, and Treg cell levels were higher in the high-risk group, while aDC, DC, macrophage, NK cell, pDC, and Tfh, Th2, and Treg cell levels were high in the high-risk group in the ICGC cohort. However, mast cells and NK cells in the TCGA cohort and NK cells in the ICGC cohort showed an inverse tendency (Figure S2A,B). As presented in Figure S2C,D, most immune-related pathways were upregulated in the high-risk group except for Type I IFN Response and Type II IFN Response.

**Figure 6:**
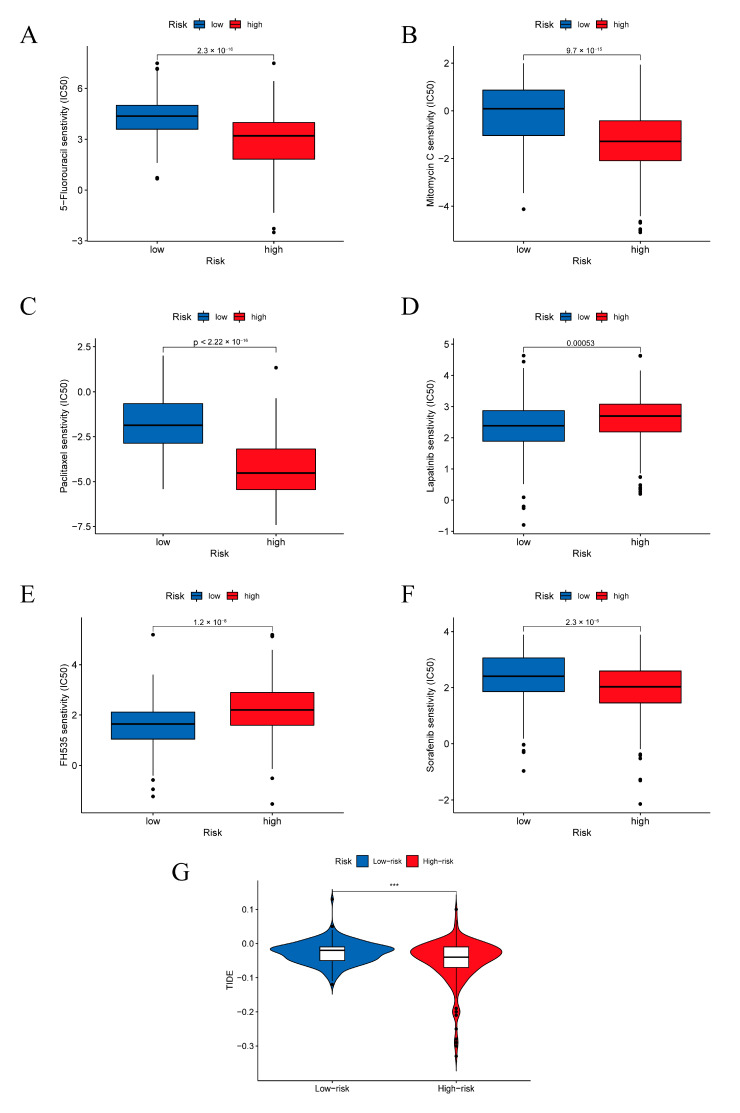
Evaluation of treatment responsiveness in the TCGA cohort. Responsiveness to the chemical drugs 5-fluorouracil (**A**), mitomycine C (**B**), and paclitaxel (**C**) in the TCGA cohort. Responsiveness to the targeted drugs lapatinib (**D**), FH535 (**E**), and sorafenib (**F**) in the TCGA cohort. (**G**) Tide scores of patients in the TCGA cohort. *** *p* < 0.001.

We evaluated treatment responsiveness to explore whether the lactylation-related prognostic model could be used to guide the treatment of HCC patients. Numerous chemical and targeted drugs were analyzed in this study. We found that when treating patients in the high-risk group, the IC50 values of chemical drugs used in chemotherapy such as 5-fluorouracil, mitomycin C, and paclitaxel were significantly lower than those in the low-risk group (Figure 6A–C). This finding indicates that patients in the high-risk group may benefit more from these chemical drugs than patients in the low-risk group. For targeted small-molecule inhibitors, the results showed that patients in the low-risk group were more sensitive to FH535 and lapatinib, while sorafenib had a lower IC50 in the high-risk group (Figure 6D–F). TIDE is widely used to evaluate responsiveness toward immunotherapy. A higher TIDE value indicates a more effective response to immunotherapy. Figure 6G shows that patients in the high-risk group responded more poorly to immunotherapy than those in the low-risk group.

### 2.7. Signature Gene Structure Analysis and Mutation Distribution

**Figure 7:**
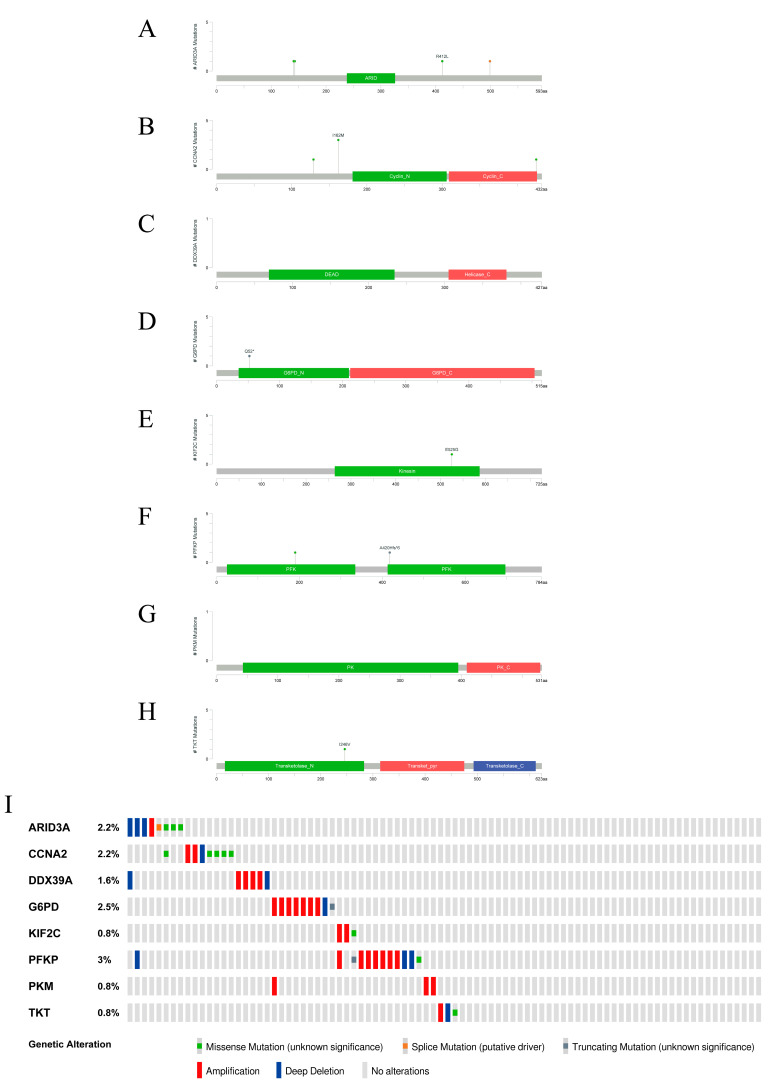
Signature gene structure and mutation distribution. (**A**–**H**) The structural domains of each gene: ARID3A/DRIL1, CCNA2, DDX39A/DDX39, G6PD, KIF2C/KNSL6, PFKP, PKM, and TKT. (**I**) The aggregate mutation conditions of the eight genes.

To further explore the possible influence of the lactylation modification of each gene from the signature, we employed liver data from the cBioPortal database to identify the structural domains and the mutation conditions of the genes. The structural domain of each gene is shown in Figure 7A–H. Figure 7I shows the aggregate mutation conditions of the eight genes: ARID3A/DRIL1, CCNA2, DDX39A/DDX39, G6PD, KIF2C/KNSL6, PFKP, PKM, and TKT.

### 2.8. Prognostic Investigation of the Glycolysis Rate-Limiting Enzyme PKM2 

**Figure 8:**
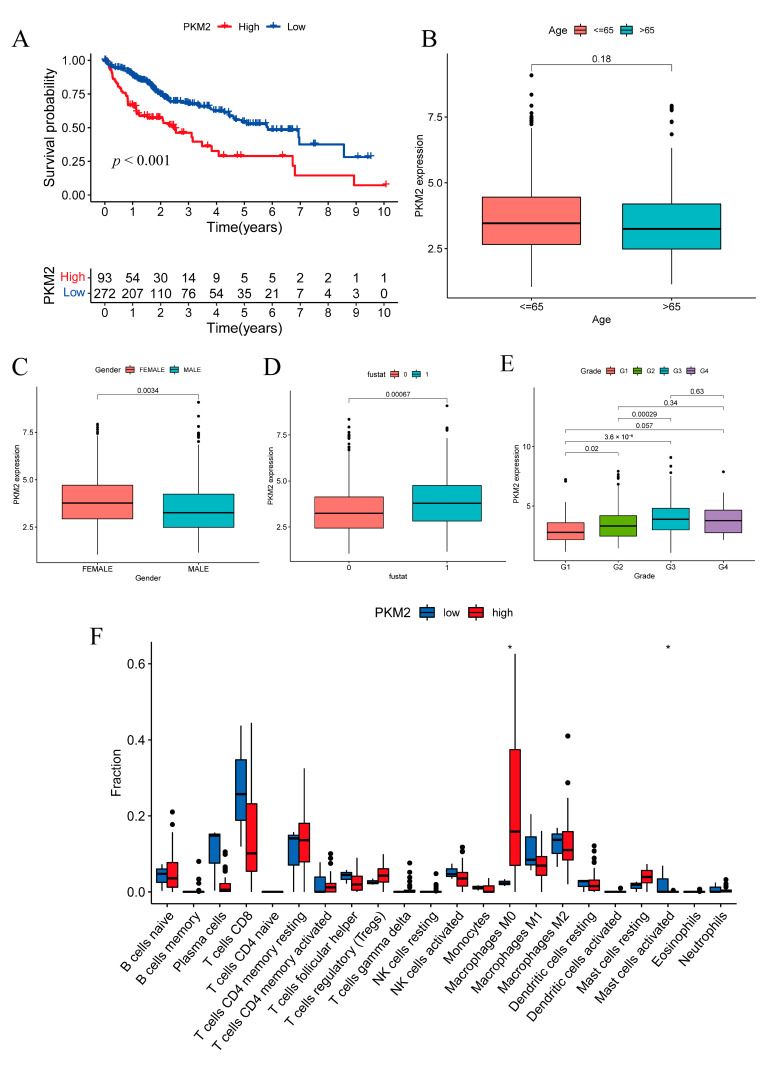
Correlations between PKM2 expression, clinical characteristics and immune cell infiltration. (**A**) HCC OS was correlated with the PKM2 expression levels in the TCGA cohort. (**B**) Patient age showed no significant correlation with the PKM2 expression level. (**C**) Female patients tended to have higher PKM2 expression levels than male patients. (**D**) Patient survival state correlated with PKM2 expression level (fustat 0: alive, 1: dead). (**E**) Patient grades partially correlated with the PKM2 expression level (Grades 1, 2, 3) except for Grade 4. (**F**) Immune cell infiltration analysis indicated that there were differences in the M0 macrophages and activated mast cells between the PKM2 high and low subgroups (The bullets in the figure show the statistical outliers in the data). * *p* < 0.05.

PKM2 is a prognostic lactylation-related DEG that is part of the gene signature. Based on its expression level, patients in the TCGA cohort (N = 365) could be divided into a high-risk group (N = 93) and a low-risk group (N = 272) (Figure 8A), indicating that high PKM2 expression was related to worse prognosis. The relevance of PKM2 expression and the resulting clinical characteristics were evaluated. PKM2 expression was irrelevant to patient age (threshold 65 years old) (Figure 8B). Female sex was correlated with higher PKM2 expression (Figure 8C). The survival condition was also correlated with PKM2 expression, and high PKM2 expression was associated with death (Figure 8D). There were also correlations between certain HCC grades and PKM2 expression (Figure 8E). The analysis of immune cell infiltration also indicated that there were differences in M0 macrophages and activated mast cells between the PKM2 high and low subgroups (Figure 8F). 

## 3. Discussion

In this study, we investigated prognostic lactylation-related genes and researched both glycolysis and the TIME in the HCC samples. Ultimately, eight genes were identified and a prognostic model was constructed. The prognostic gene signature clearly divided the HCC patients into two groups and could effectively predict treatment responsiveness and clinical outcome. The prognostic role played by PKM2 was also investigated.

HCC is the fifth most prevalent cancer in the world [19]. Many studies have noted that metabolic reprogramming is an important characteristic of HCC occurrence and development [20,21,22,23]. The GSEA enrichment results in our study confirmed that the glycolysis pathway was abnormally elevated in the tumor tissues. Excessive lactate accumulation leads to widespread protein lactylation. Lactylation modification was initially found to occur on histones. Yang and coworkers conducted immunostaining in HCC tissues and observed widespread protein lactylation. They further conducted mass spectrometry analysis and confirmed that lactylation largely occurred on both histones and nonhistone proteins [14]. Protein lactylation has been found to be related to cell metabolism and cancer immune regulation [24]. 

The TCGA database is a cancer genomics program with genomic, transcriptomic, and epigenomic data for over 30 types of cancer. The ICGC database contains data on 50 cancer types and subtypes. The transcriptomic data of the cancer patients on these databases are provided with the clinical treatment and prognosis. The TCGA and ICGC databases are widely used to reveal the influence of oncogene mutation and modification, define cancer subtypes, and benefit cancer prognosis and therapeutic management [25]. Both glycolysis and the TIME in the HCC samples were researched in this study. The TIME is a dynamic system consisting of cancer cells, extracellular matrix, stromal cells, and cytokines [26,27]. Targeting the TIME is crucial in overcoming tumor immune escape, potentiating host antitumor immune responses, and enhancing the efficacy of HCC combination immunotherapies [28]. In our research, the two subgroups in both the TCGA and ICGC cohorts exhibited major differences in immune cell abundance and immune pathway intensity, suggesting that a major immune disorder may exist in the high-risk group. The GO analysis in the TCGA cohort also showed strong relevance with the immune pathways. Wang et at. found that the lactylation of PKM2 could suppress inflammatory metabolic adaptation in proinflammatory macrophages [16]. Lactylation of other proteins may interact with the TIME in similar ways. In addition, drug responsiveness evaluations revealed that the patients in different subgroups exhibited different responses to chemical and targeted drugs, with patients in the high-risk group exhibiting more sensitivity to most chemical drugs and certain targeted drugs including sorafenib. These results demonstrate that the gene signature is helpful for clinical HCC treatment (Figure S4).

Ultimately, eight genes were identified and formed the prognostic gene signature: ARID3A/DRIL1, CCNA2, DDX39A/DDX39, G6PD, KIF2C/KNSL6, PFKP, PKM, and TKT. AT-rich interaction domain 3A (ARID3A) is a DNA binding protein. This transcription factor has been reported to be a transcriptional activator that promotes megakaryocytic differentiation in concert with GATA1 [29]. Shen et al. reported that ARID3A can promote liver cancer cell viability and metastasis [30]. Cyclin A2 (CCNA2) encoded protein belongs to the cyclin family and functions as a regulator of the cell cycle. It has been researched in pancreatic and colorectal cancers and has been found to be an independent prognostic factor of colorectal tumors [31,32]. DExD-box helicase 39A (DDX39A) is a putative RNA helicase. Kinesin family member 2C (KIF2C) is an M-kinesin and is essential for mitosis [33]. Shi et al. reported that KIF2C promoted HCC cell proliferation, migration, invasion, and metastasis [34].

G6PD, PFKP, PKM2, and TKT are all important enzymes involved in energy metabolism. Glucose-6-phosphate dehydrogenase (G6PD) is a key regulatory enzyme in the pentose phosphate pathway. It produces nicotinamide adenine dinucleotide phosphate (NADPH) and helps maintain the reducing environment in cells [35]. It has been reported that high G6PD expression increases doxorubicin resistance in triple negative breast cancer and is associated with a reduction in progression-free survival in prostate cancer bone metastasis [36]. TKT participates in the Calvin cycle and the pentose phosphate pathway. Qin and colleagues found that TKT promotes HCC development in a nonmetabolic manner related to nuclear localization and the EGFR pathway [37]. It has also been reported that reduced expression of TKT and CTPS regulates flux into pyrimidine biosynthesis, which correlates with better prognosis in pancreatic cancer [38]. 

Phosphofructokinase platelet (PFKP) and pyruvate kinase M2 (PKM2) are two key glycolysis rate-limiting enzymes. PFKP is the platelet isoform of PFK1, which catalyzes the step of converting fructose 6-phosphate and ATP to fructose 1,6-bisphosphate and ADP [39]. It has been broadly reported that PFKP expression is elevated in many cancers and is correlated with worse prognosis in breast cancer, leukemia, lung cancer, and HCC [40,41,42,43]. PKM2 catalyzes the dephosphorylation of phosphoenolpyruvate to pyruvate. Tetrameric and dimeric forms can be formed by PKM2, and the PKM2 dimer state can enter the nucleus to regulate gene expression. This transformation is crucial in tumor cell anaerobic glycolysis and energy supply [44]. In this research, we confirmed that elevated PKM2 expression in tumor cells correlated with clinical outcome, and there were also major differences in the TIME of the two subgroups defined by PKM2 expression. Wang et al. investigated the lactylation of PKM2 and confirmed that PKM2 lactylation could suppress inflammatory metabolic adaptation in proinflammatory macrophages. This finding confirmed the result of our analysis, which indicated that the modification site K62 on PKM2 was in the structural domain and may affect the function of the protein [16]. 

Although the lactylation-related signature has been validated by multidimensional methods, there were still limitations to this study. As research in the protein lactylation field is still in its preliminary stages, how lactylation modification affects protein function in tumor cells remains to be illustrated. Because of the lack of corresponding lactylation antibodies, the experimental validation of all signature genes currently faces practical problems. However, we believe that these issues will be solved as we gain a better understanding of lactylation in the near future. For future research in the field, lactylation proteomic studies will be essential for expanding the novel lactylation gene pool. It will also be useful to integrate protein expression data with the corresponding lactylation modification data. Additionally, it is recommended to develop specific lactylation modification protein antibodies and use these to study the functional effects of lactylation on proteins and identify the underlying mechanism.

## 4. Materials and Methods

### 4.1. Data Acquisition

The mRNA transcriptome profiles and corresponding clinical information of 365 patients were downloaded from The Cancer Genome Atlas (TCGA) database, https://protal.gdc.cancer.gov/ (accessed on 20 January 2023). External validation data with 231 patients were obtained from the Japanese HCC patient cohort LIRI-JP from The International Cancer Genome Consortium (ICGC) database, https://dcc.icgc.org/ (accessed on 20 January 2023). A scale method-based normalization was performed with the gene expression profiles using the R package “limma” (v3.54.0). The database guidelines were strictly followed when using these data.

The lactylation-related genes include one lactylase EP300 and six delactylases, namely, HDAC1-3 and SIRT1-3, according to previously published studies [5,45]. Wan et al. reported a novel diagnostic ion-based strategy and identified various lactylation sites in human cell lines. A total of 327 identified lactylated proteins were included in our research. A total of 332 lactylation-related genes were included and are presented in Supplementary Table S1.

### 4.2. Identification of Differentially Expressed and Prognostic Genes

The mRNA data from the TCGA and ICGC databases were extracted using the R package “limma”. The expression profiles of the differentially expressed genes (DEGs) and prognostic lactylation-related genes were analyzed, and intersectional genes were identified. The criteria for DEG identification were a false discovery rate (FDR) < 0.05 and |log2 FC| ≥ 2. By applying these criteria, genes expressed at more than 4-fold levels in tumor tissues and adjacent tissues were screened out at a false discovery rate of less than 0.05. The criteria were set to best identify statically significant differentially expressed genes. Clinical information was integrated with expression data using the R package “bioconductor” (v3.16). Univariate Cox analysis was performed with a threshold of *p* < 0.05, and prognosis-related genes were screened out. The Venn diagram was generated by the R package “venn” (v1.11). We then used the R package “pheatmap” (v1.0.12) to construct an expression heatmap. A forest map showing *p* values and hazard ratios (HRs) was generated with the R package “survival” (v3.4.0). The protein-protein interaction (PPI) and correlation network analyses were conducted via the STRING database and the R packages “corrplot” (v0.92) and “circlize” (v0.4.15), respectively. 

### 4.3. Analysis of Glycolysis Pathway Enrichment

Gene set enrichment analysis (GSEA) was carried out to analyze the enrichment of the glycolysis pathway. Full expression data of genes in both cancer samples and adjacent samples were obtained in previous procedures. The glycolysis gene set was retrieved from the GSEA database, https://www.gsea-msigdb.org/gsea/ (accessed on 20 January 2023). The number of simulations was set to 1000. The analysis was conducted by GSEA (4.3.2).

### 4.4. Construction and Validation of a Prognostic Lactylation-Related Gene Signature

The gene signature was generated by the R packages “glmnet” (v4.1.6) and “survival”. Univariate Cox analysis of the overall survival (OS) of the lactylation-related DEGs was conducted. TCGA cohort cancer samples were used as the training dataset. LASSO regression was utilized to search for a suitable potential gene set for the prognostic signature. The optimal penalty parameter λ was generated to determine the gene coefficient of the risk score formula. The risk score model trained from the TCGA data was constructed as follows:

$$Riskscore=\sum_{i=1}^{N}\left(\exp\times coef\right)$$

where *N* is the number of model genes; exp represents the expression value of genes; coef is the coefficient of each gene. Patients from the TCGA database were divided into two groups, and Kaplan-Meier analysis was conducted to evaluate differences in overall survival (OS) time between the two groups. The receiver operating characteristic (ROC) curve was generated using the R package “timeROC” (v0.4) to evaluate the sensitivity and specificity of the risk model. The ICGC cohort was used in the validation of the gene signature.

### 4.5. Functional Annotation Analysis

Wilcoxon tests were conducted to identify differentially expressed genes in the two groups divided by risk scores using the R package “limma” with criteria of a FDR < 0.05 and |log2 FC| ≥ 1. Gene Ontology (GO) enrichment analyses were conducted using the R packages “colorspace” (v2.0.3), “stringi” (v1.7.8), and “ggplot2” (v3.4.0). Single-sample gene set enrichment analysis (ssGSEA) was carried out to quantify immune pathway enrichment in the cancer samples. The immune cell infiltration abundance levels were evaluated through standardization of the gene expression levels and enrichment scores calculated by the empirical cumulative distribution function. These processes were realized with the R packages “BiocManager” (v1.30.19), “limma”, “GSVA” (v1.46.0) and “GSEABase” (v1.60.0).

### 4.6. Treatment Responsiveness Evaluation

The treatment responsiveness evaluations consisted of drug sensitivity and immunotherapy efficacy analyses. The drug sensitivity analysis was conducted using the R package “pRRophetic” (v.0.5). The major rationale of this methodology involves building statistical models from the gene expression data obtained from an enormous panel of cancer cell lines and then applying the models to the target sample gene expression data [46]. The IC50 values of various therapeutic agents in the two groups were calculated, and drugs with different IC50 values (*p* < 0.001) were displayed with box plots. The immune dysfunction and exclusion (TIDE) scoring was conducted on the website (http://tide.dfci.harvard.edu/, accessed on 20 January 2023) and then analyzed using the R packages “limma” and “ggpubr”. TIDE is a framework used to evaluate the potential of tumor immune escape based on tumor gene expression data. Vast amounts of tumor sample omics data are processed, and information on T-cell dysfunction is employed to predict patient survival. T-cell dysfunction was evaluated by researching the correlations between cytotoxic T lymphocyte infiltration and the expression of each gene in the tumor tissues [47,48].

### 4.7. Mutation Analysis of Signature Genes 

Mutation analysis of the genes comprising the gene signature was conducted on the cBioPortal database, http://www.cbioportal.org/ (accessed on 20 January 2023), and the structural domain of each gene was also obtained to evaluate the possible effect of lactylation modification. Liver data from the TCGA database were used in the analysis.

### 4.8. Survival Analysis and Clinical Relevance Analysis for Single Gene

The patients in the TCGA cohort were divided into two subgroups according to single-gene expression levels using the R packages “survival” and “survminer” (v0.4.9). Then, the relevance between the clinical characteristics and target gene expression levels was analyzed and shown in box plots using the R package “ggpubr” (v0.4.0).

### 4.9. Tumor-Infiltrating Immune Cell Profiling

To evaluate the tumor immune microenvironment (TIME), we utilized the CIBERSORT algorithm in the two groups. CIBERSORT is a machine-learning algorithm for the high-throughput characterization of different cell types [49]. The following R packages were used: “preprocessCore” (v1.52.1), “e1071” (v1.7.9), “limma”, “ggpubr”, “vioplot” (v0.4.0), “ggExtra” (v0.10.0), and “reshape2” (v1.4.4).

### 4.10. Statistical Analysis

The PCA and t-SNE analysis were applied using the R packages “Rtsne” (v0.16) and “ggplot2” (v3.4.0). Univariate and multivariate Cox regression analyses were conducted using the R package “survival”. All statistical analyses were conducted based on R (v4.0.2). Statistical significance is indicated with asterisks (*). A two-sided *p* value of <0.05 was considered as statistically significant (* *p* < 0.05, ** *p* < 0.01, *** *p* < 0.001).

## 5. Conclusions

We identified prognostic DEGs and developed a prognostic gene signature in HCC based on lactylation-related genes. The glycolysis pathway abundance and TIME were also analyzed. The gene signature provided a practical drug responsiveness evaluation for clinical treatment. In conclusion, this research provides a practical lactylation-based gene signature to predict HCC prognosis and a new perspective for lactylation modification studies.

## Figures and Tables

**Table 1:** Clinical and pathological characteristics of the HCC patients retrieved from the TCGA and ICGC databases.

Characteristics		TCGA Cohort	ICGC LIRI-JP Cohort
Total number of patients		365	231
Survival status	Survival	235 (64.38%)	189 (81.82%)
	Death	130 (35.62%)	42 (18.18%)
Age	≤65 years	227 (62.19%)	89 (38.53%)
	>65 years	138 (37.81%)	142 (61.47%)
Sex	Male	246 (67.40%)	170 (73.59%)
	Female	119 (32.60%)	61 (26.41%)
Stage	I	170 (46.58%)	36 (15.58%)
	II	84 (23.01%)	105 (45.45%)
	III	83 (22.74%)	71 (30.74%)
	IV	4 (1.10%)	19 (8.23%)
	Unknow	24 (6.57%)	0
Pathological grade (Edmondson)	G1	55 (15.07%)	20 (8.66%)
	G2	175 (47.94%)	134 (58.01%)
	G3	118 (32.33%)	56 (24.24%)
	G4	12 (3.29%)	1 (0.43%)
	Unknow	5 (1.37%)	20 (8.66%)
Metastasis	Primary HCC	365	201 (87.01%)
	Metastatic HCC	0	30 (12.99%)

## Data Availability

Data is contained within the article and supplementary material.

## Supplementary Materials

The following supporting information can be downloaded at: https://www.mdpi.com/article/10.3390/ph16050644/s1. Supplementary Table S1. List of 332 lactylation-related genes. Supplementary Table S2. Prevalence of hepatitis viral infection in the TCGA cohort. Supplementary Table S3. TCGA hepatitis viral infection prevalence. Supplementary Figure S1. Validation of the prognostic signature in the ICGC cohort. Supplementary Figure S2. Results of the TIME landscape analysis in the TCGA and ICGC cohorts. Supplementary Figure S3. The AFP expression level was significantly higher in the high-risk group than in the low-risk group (*** p < 0.001). Supplementary Figure S4: High-risk and low-risk group according to the risk score calculated by the gene signature.

## Abbreviations

HCC: hepatocellular carcinoma; PD-1: programmed death-1; CTLA-4: cytotoxic T-lymphocyte-associated protein 4; ICI: immune checkpoint inhibition; DEG: differentially expressed gene; HR: hazard ratio; OS: overall survival; LASSO: least absolute shrinkage selection operator; TIME: tumor immune microenvironment; PCA: principal component analysis; t-SNE: t-distributed stochastic neighbor embedding; ROC: receiver operating characteristic; AUC: area under the curve.
