# Supplementary material for: Lactylation-Related Gene Signature Effectively Predicts Prognosis and Treatment Responsiveness in Hepatocellular Carcinoma
Source: Pharmaceuticals (Basel). 2023 Apr 25;16(5):644. doi: 10.3390/ph16050644 (PMC10221268; doi:10.3390/ph16050644)
Supplement: Supplementary file 1 [file pharmaceuticals-16-00644-s001.zip › Supplementary Figures.pdf]

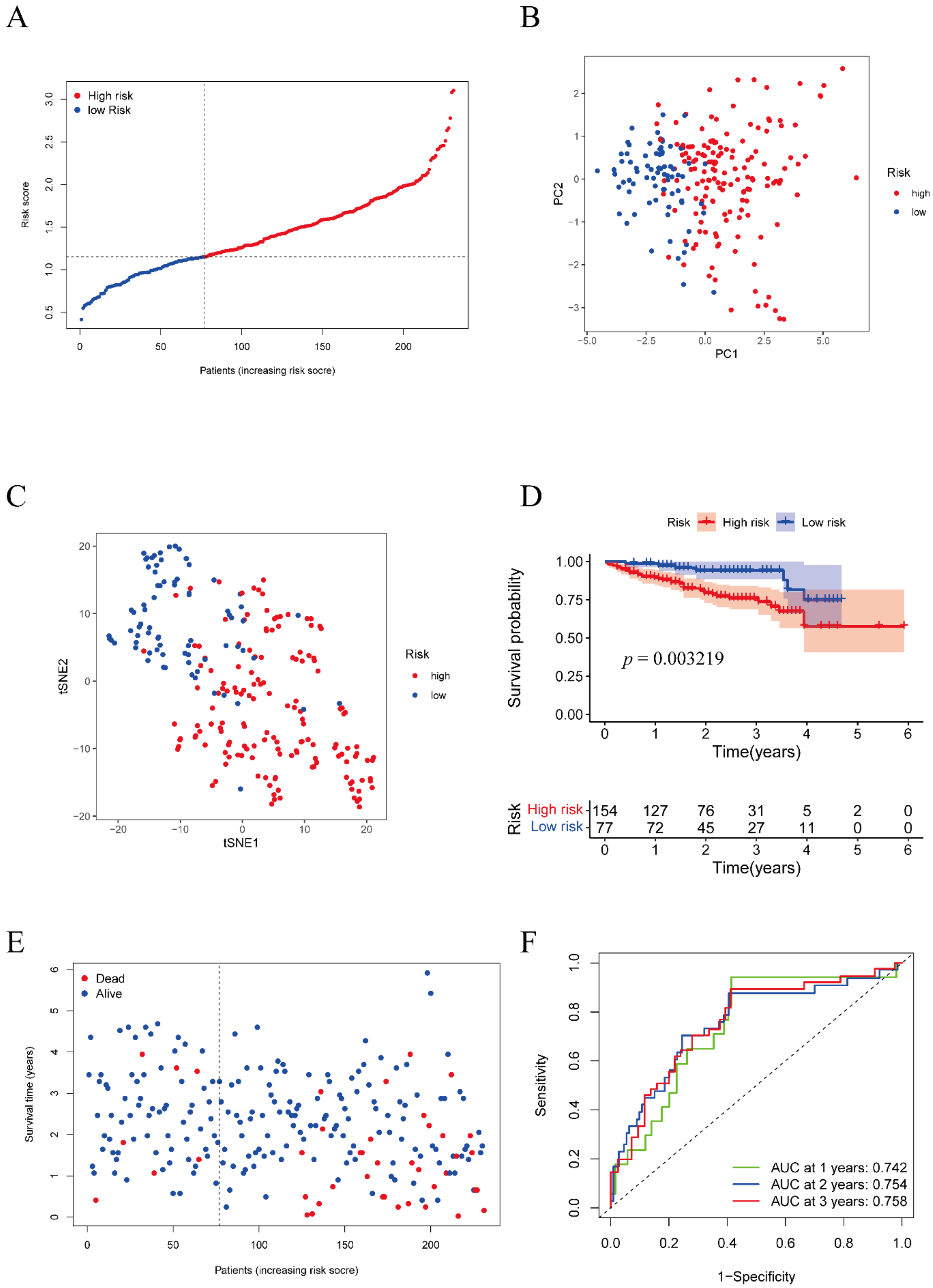
A
B
C
D
E
F
High risk
low Risk
Risk score
Patients (increasing risk socre)
PC1
PC2
Risk
high
low
tSNE1
tSNE2
Risk
high
low
Risk
High risk
Low risk
Survival probability
p = 0.003219
Time(years)
Risk
High risk 154 127 76 31 5 2 0
Low risk 77 72 45 27 11 0 0
Time(years)
Dead
Alive
Survival time (years)
Patients (increasing risk socre)
Sensitivity
1−Specificity
AUC at 1 years: 0.742
AUC at 2 years: 0.754
AUC at 3 years: 0.758

Figure S1. Validation of the prognostic signature in the ICGC cohort.(A) Distribution of the risk score in the ICGC cohort.(B) PCA analysis of patients in ICGC cohort.(C) T-SNE analysis of patients in ICGC cohort.(D) Kaplan-Meier survival curve of HCC overall survival in the ICGC cohort.(E) Distribution of survival status with increasing risk score in the ICGC cohort.(F) ROC curve showed that the 8-gene prognostic signature had satisfactory predictive efficacy in the ICGC cohort.

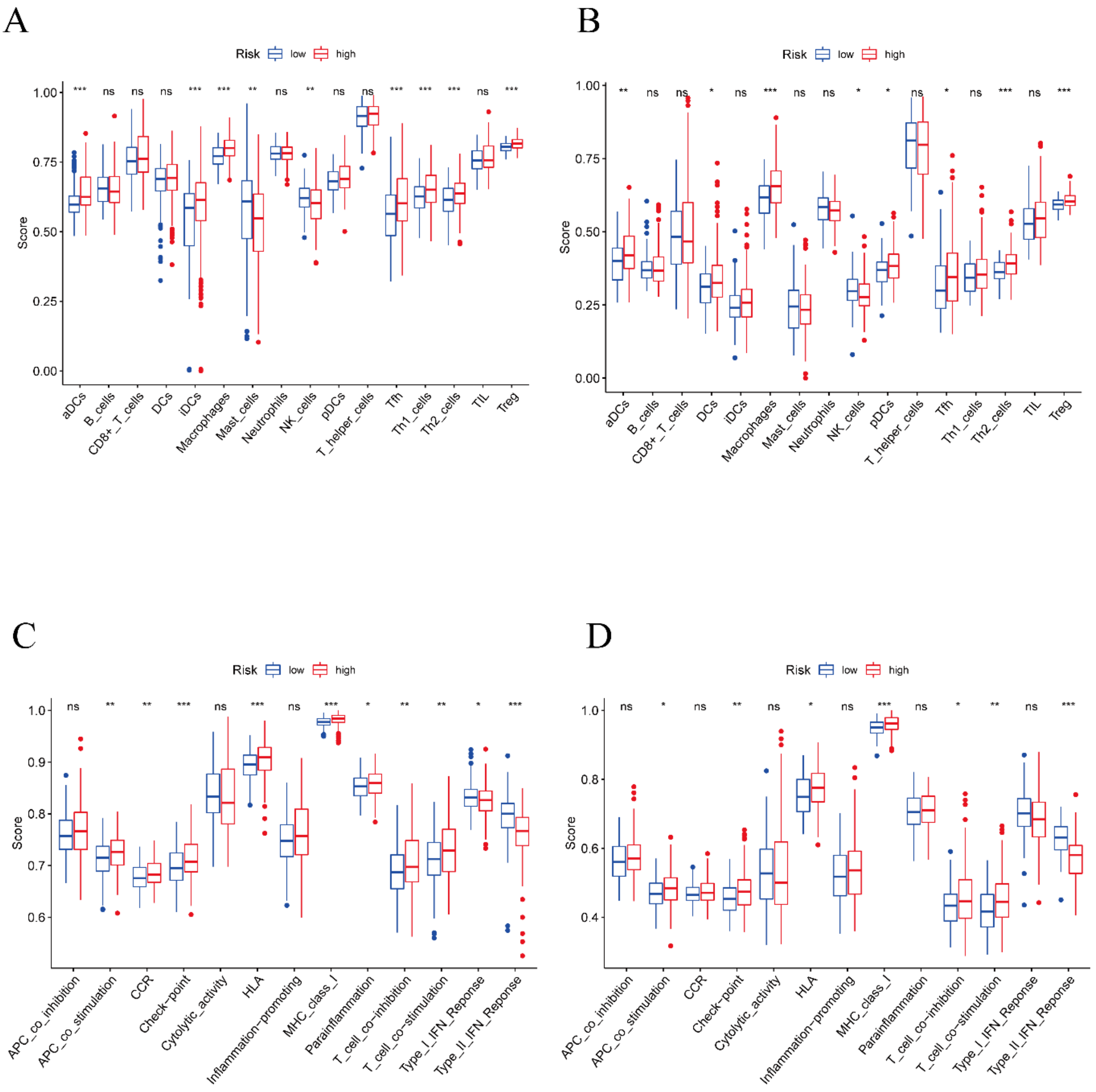
A
B
C
D
Risk
low
high
Score
1.00
0.75
0.50
0.25
0.00
aDCs
B_cells
CD8+_T_cells
DCs
iDCs
Macrophages
Mast_cells
Neutrophils
NK_cells
pDCs
T_helper_cells
Tfh
Th1_cells
Th2_cells
TIL
Treg
1.0
0.9
0.8
0.7
0.6
0.4
APC_co_inhibition
APC_co_stimulation
CCR
Check-point
Cytolytic_activity
HLA
Inflammation-promoting
MHC_class_I
Parainflammation
T_cell_co-inhibition
T_cell_co-stimulation
Type_I_IFN_Reponse
Type_II_IFN_Reponse

Figure S2. Results of TIME landscape analysis in the TCGA and ICGC cohorts.(A) The abundance of immune cells in the TCGA cohort.(B) The abundance of immune cells in the ICGC cohort.(C) Immune function pathway analysis in the TCGA cohort.(D) Immune function pathway analysis in the ICGC cohort. (*$p < 0.05$, **$p < 0.01$ and ***$p < 0.001$.).

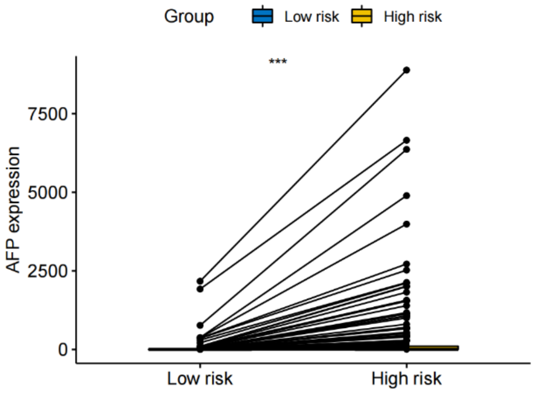

Figure S3. The AFP expression level was significantly higher in the high-risk group than in the low-risk group (*** $p < 0.001$).

Gene Signature in Colorectal Cancer Patients

To test if the gene signature can be used to predict prognosis of other cancer patients, we obtained mRNA transcriptome profile of colorectal cancer (CRC) samples in TCGA cohort (372 patients) and analyzed the gene expression level and clinical outcome data. Subsequently the patients were divided into two groups by the risk score calculated by the gene signature established in HCC patients. Result showed that there was no significant difference of overall survival between the high-risk and low-risk group (p = 0.96). The conclusion is that the gene signature can not be used in CRC patients.

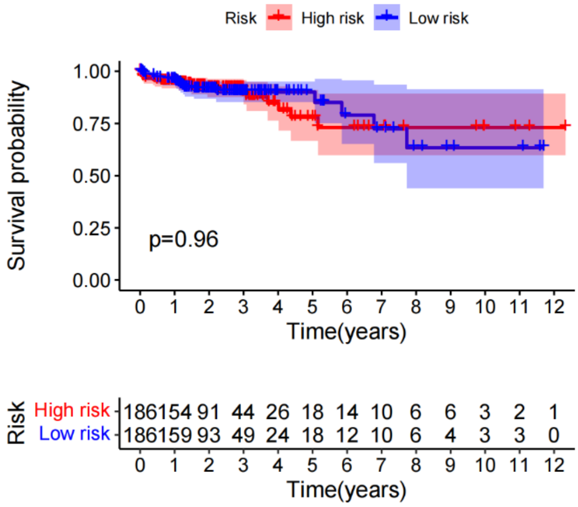

Figure S4. High-risk and low-risk group according to the risk score calculated by the gene signature.
