# Supplementary material for: Lactylation-Related Gene Signature Effectively Predicts Prognosis and Treatment Responsiveness in Hepatocellular Carcinoma
Source: Pharmaceuticals (Basel). 2023 Apr 25;16(5):644. doi: 10.3390/ph16050644 (PMC10221268; doi:10.3390/ph16050644)
Supplement: Supplementary file 1 [file pharmaceuticals-16-00644-s001.zip › Table S2 Prevalence of hepatitis viral infection in the TCGA cohort.pdf]

Table S2. Prevalence of hepatitis viral infection in TCGA cohort.

		TCGA cohort
Hepatitis viral infection	HBV	223 (61.10%)
	HCV	133 (36.44%)
	HBV and HCV	84 (23.01%)
	Uninfected	93
Total number of patients		365
